# Supplementary material for: Factors Affecting the Population of Excited Charge Transfer States in Adenine/Guanine Dinucleotides: A Joint Computational and Transient Absorption Study
Source: Biomolecules. 2024 Dec 3;14(12):1548. doi: 10.3390/biom14121548 (PMC11673769; doi:10.3390/biom14121548)
Supplement: Supplementary file 1 [file biomolecules-14-01548-s001.zip › biomolecules-3281336-supplementary.pdf]

Factors affecting the population of excited charge transfer states in adenine/guanine dinucleotides: a joint computational and transient absorption study

Vasilis Petropoulos [1], Lara Martinez-Fernandez* [2], Lorenzo Uboldi [1], Margherita Maiuri [1], Giulio Cerullo* [1,3], Evangelos Balanikas [4] and Dimitra Markovitsi* [5]

[1] Dipartimento di Fisica, Politecnico di Milano, Piazza Leonardo da Vinci 32, 20133 Milano, Italy; lorenzo.uboldi@polimi.it (L.U.)
[2] Departamento de Química Física de Materiales, Instituto de Química Física Blas Cabrera, Consejo Superior de Investigaciones Científicas, Calle Serrano 119, 28006 Madrid, Spain
[3] Istituto di Fotonica e Nanotecnologie-CNR, Piazza Leonardo da Vinci 32, 20133 Milano, Italy
[4] Laboratoire d'Optique et Biosciences, Ecole Polytechnique, CNRS—INSERM, Institut Polytechnique de Paris, 91120 Palaiseau, France
[5] Institut de Chimie Physique, CNRS-UMR8000, Université Paris-Saclay, 91405 Orsay, France
* Correspondence: lmartinez@iqf.csic.es (L.M.-F.); giulio.cerullo@polimi.it (G.C.); dimitra.markovitsi@universite-paris-saclay.fr (D.M.)

Supplementary Materials

- **Figures with experimental results**

- **Tables with computational results**

➢ Figures with experimental results

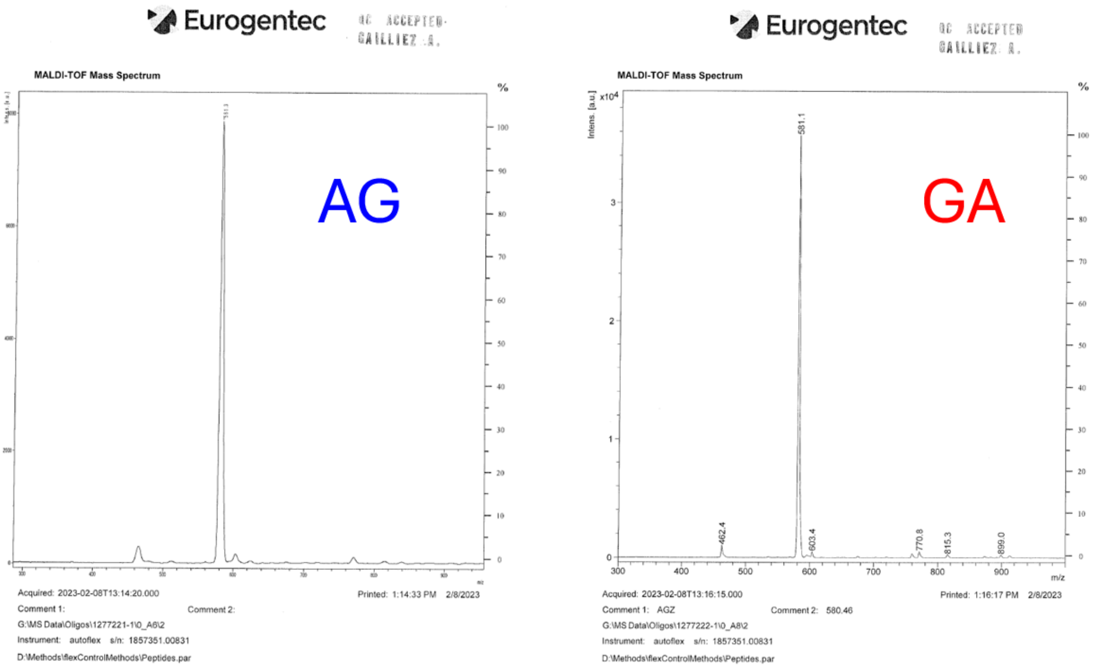

Figure S1. MALDI-TOF spectra provided by Eurogentec for the studied samples.

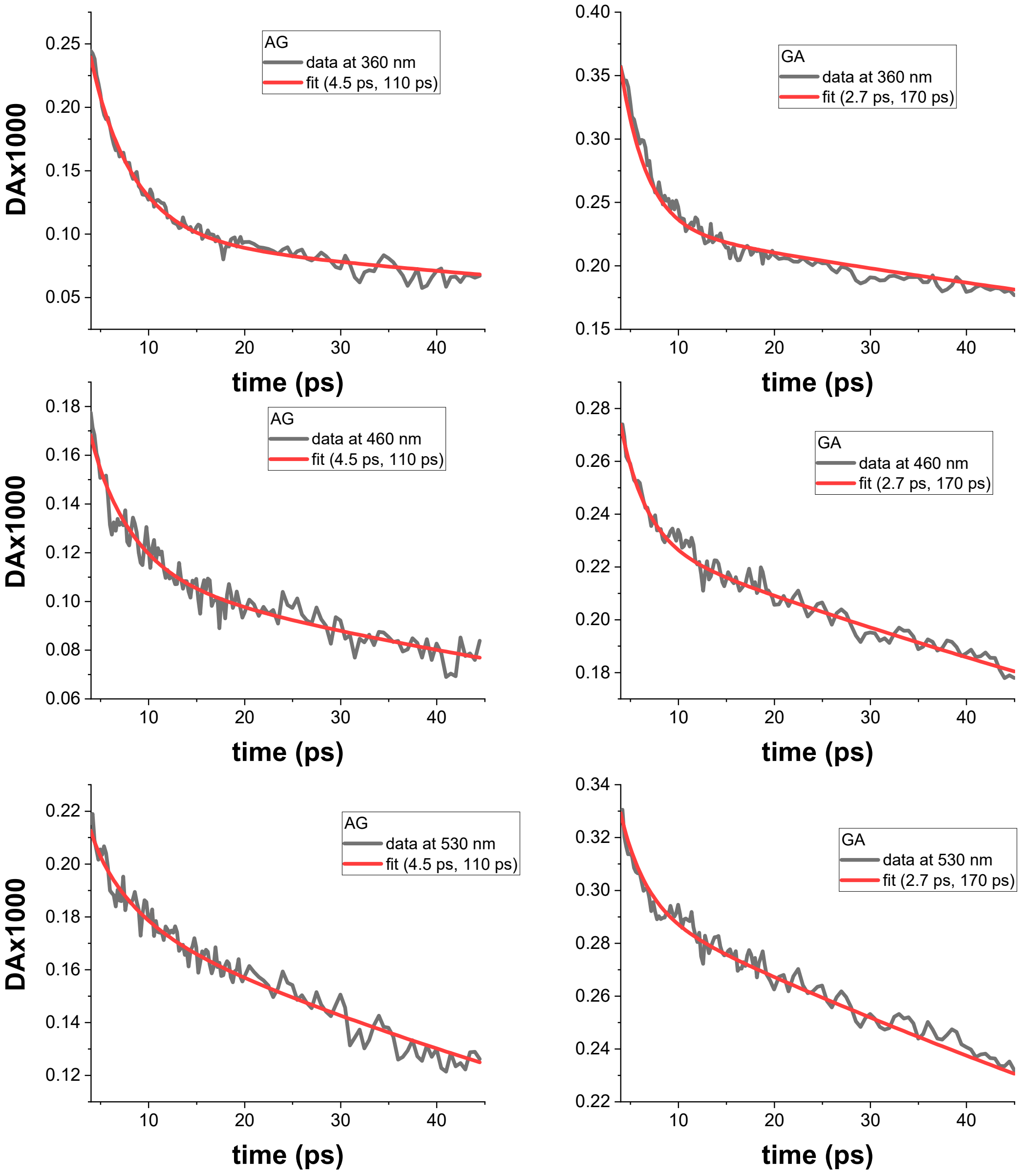

Figure S2. Fits of the *TA* decays between 4 and 40 ps with two exponential functions, one of which was fixed to 112 ps for **AG** and 170 ps for **GA**.

Tables with computational results

Table S1. Relative energies of the different possible **AG** conformers with respect to the *anti-anti* one, computed at the PCM/M052X/6-31G(d) level of theory.

conformer	ΔE in eV (kcal/mol)	ΔG in eV (kcal/mol)
anti-anti	0.0 (0.0)	0.0 (0.0)
anti-syn	-0.08 (-1.9)	-0.05 (-1.1)
syn-anti	0.03 (0.7)	0.06 (1.3)
syn-syn	0.10 (2.4)	0.10 (2.2)

Table S2. Relative energies of the different possible **GA** conformers with respect to the *anti-anti* one, computed at the PCM/M052X/6-31G(d) level of theory.

conformer	ΔE in eV (kcal/mol)	ΔG in eV (kcal/mol)
anti-anti	0.0 (0.0)	0.0 (0.0)
anti-syn	0.14 (3.3)	0.11 (2.7)
syn-anti	0.48 (11.1)	0.25 (5.7)
syn-syn	0.33 (7.5)	0.30 (7.1)

Table S3. Properties of the Franck-Condon states determined for *anti-anti* **AG** at the PCM/M052X/6-31G(d) level of theory. VAE: vertical absorption energy; f: oscillator strength; δ: charge transfer character [1].

	character	VAE (eV)	f	δ (a.u.)
S_1	ππ*G(La)	5.30	0.133	0.0
S_2	ππ*A	5.48	0.171	0.0
S_3	ππ*A	5.57	0.008	0.0
S_4	G→A *CT*	5.61	0.198	0.6
S_5	nπ*G	5.62	0.005	0.0
S_9	G→A *CT*	6.35	0.008	0.9
S_{10}	A→G *CT*	6.40	0.004	0.8

Table S4. Properties of the Franck-Condon states determined for *anti-syn* **AG** at the PCM/M052X/6-31G(d) level of theory. VAE: vertical absorption energy; f: oscillator strength; δ: charge transfer character.

	character	VAE (eV)	f	δ (a.u.)
S_1	ππ*G(La)	5.25	0.116	0.01
S_2	ππ*A	5.50	0.337	0.00
S_3	nπ*A	5.59	0.010	0.00
S_4	nπ*G	5.62	0.014	0.01
S_5	ππ*A	5.67	0.022	0.00
S_7	G→A *CT*	5.96	0.008	0.86

Table S5. Properties of the Franck-Condon states determined for *anti-anti* **GA** at the PCM/M052X/6-31G(d) level of theory. VAE: vertical absorption energy; f: oscillator strength; δ: charge transfer character [1].

	character	VAE (eV)	f	δ (a.u.)
S_1	ππ*G(La)	5.30	0.020	0.0
S_2	ππ*A + CT	5.38	0.179	0.3
S_3	ππ*A + CT	5.51	0.267	0.1
S_4	nπ*A	5.55	0.050	0.1
S_5	ππ*A	5.60	0.010	0.0
S_9	G→A *CT*	6.26	0.014	0.8

Table S6. Properties of the minima located on the *PES* of the first excited state determined for *anti-anti* **AG** at the PCM/M052X/6-31G(d) level of theory. ADE: adiabatic (with respect to the S_0 at the FC region) energies; VEE: vertical (with respect to the S_0 at the corresponding minima) emission energies; f: oscillator strength; initial state optimized and main reaction coordinate. REF

	ADE (eV)	VEE (eV)	f	initial state	Reaction coordinate
min-ππ*G (La)	4.61	2.68	0.041	S_1,S_5	C1-C2-NH2
min-*CT*	4.59	3.78	0.023	S_9	Interbase distance
min-nπ*A	4.82	4.00	0.003	S_2, S_3	Oop C2
exciton-min	4.42	2.57	0.015	S_4	^{A}C2-^{G}C2

Table S7. Properties of the minima located on the *PES* of the first excited state determined for *anti-syn* **AG** at the PCM/M052X/6-31G(d) level of theory. ADE: adiabatic (with respect to the S_0 at the FC region) energies; VEE: vertical (with respect to the S_0 at the corresponding minima) emission energies; f: oscillator strengths; initial state optimized and main reaction coordinate.

	ADE (eV)	VEE (eV)	f	initial state	Reaction coordinate
min-ππ*G (La)	4.40	2.04	0.016	S_1, S_5	C1-C2-NH2
min-*CT* (0.4 a.u.)	4.81	3.77	0.024	S_2	Interbase distance
min-nπ*A	4.81	3.48	0.010	S_3, S_4	Oop C2

Table S8. Properties of the minima located on the *PES* of the first excited state determined for *anti-anti* **GA** at the PCM/M052X/6-31G(d) level of theory. ADE: adiabatic (with respect to the S_0 at the FC region) energies; VEE: vertical (with respect to the S_0 at the corresponding minima) emission energies; f: oscillator strengths; initial state optimized and main reaction coordinate. REF

	ADE (eV)	VEE (eV)	f	initial state	Reaction coordinate
min-ππ*G (La)	4.56	2.22	0.018	S_3	C1-C2-NH2
min-*CT* (0.7 a.u.)	4.70	4.00	0.036	S_1, S_2	Interbase distance
min-nπ*A	4.72	3.39	0.007	S_4	Oop C2

1. Balanikas, E.; Martinez-Fernadez, L.; Improta, R.; Podbevsek, P.; Baldacchino, G.; Markovitsi, D., The Structural Duality of Nucleobases in Guanine Quadruplexes Controls Their Low-Energy Photoionization. *J. Phys. Chem. Lett.* **2021,** 12, (34), 8309–8313.
